# Multimorbidity in patients with low back pain in Danish chiropractic practice: a cohort study

**DOI:** 10.1186/s12998-023-00475-3

**Published:** 2023-02-10

**Authors:** Bolette Skjødt Rafn, Jan Hartvigsen, Volkert Siersma, John Sahl Andersen

**Affiliations:** 1 Research Unit for General Practice and Section of General Practice, Department of Public Health, Copenhagen University, Copenhagen, Denmark; 2 Chiropractic Knowledge Hub, Odense, Denmark; 3 Department of Sports Science and Clinical Biomechanics, University of Southern Denmark, Odense, Denmark

**Keywords:** Chiropractor, Low back pain, Multimorbidity, Recovery

## Abstract

**Background:**

People with multimorbidity, defined as the co-existence of two or more chronic conditions in an individual, often suffer from pain and functional limitations caused by musculoskeletal disorders and the chronic conditions. In chiropractic practice, two thirds of patients are treated for low back pain (LBP). It is unknown to what extent LBP is accompanied with chronic conditions in chiropractic practice. The objective was to determine the prevalence of multimorbidity among patients with LBP in chiropractric practice and to investigate if multimorbidity affects pain intensity, self-rated health, physical and mental health. Finally, to explore if individuals with multimorbidity have a different recovery for the LBP.

**Methods:**

Patients presenting with a new episode of LBP were recruited from 10 chiropractic clinics in 2016–2018. Patient-reported data concerning socio-demographics, self-rated health, pain intensity, history of LBP, mental health and chronic conditions were collected at baseline. The prevalence of multimorbidity was determined. To evaluate differences in recovery from the LBP, we estimated changes in the Roland Morris Disability Questionnaire (RMDQ) score and use of pain medication at baseline, 2 weeks, 3 months and 12 months. The analyses were adjusted using regression models.

**Results:**

2083 patients were included at baseline and 71%, 68% and 64% responded to follow-up questionnaires at 2 weeks, 3 and 12 months. 1024 (49%) participants reported to have at least one chronic condition and 421 (20%) had multimorbidity (≥ 2 chronic conditions). The presence of multimorbidity was associated with increased odds of poor self-rated health (OR 2.13), physical fitness (OR 1.79), poor muscular strength (OR 1.52), poor endurance (OR 1.51), and poor balance (OR 1.33). Patients with high LBP intensity combined with multimorbidity showed a poorer recovery than patients without chronic diseases (mean difference in RMDQ score 3.53 at 12 months follow-up). More patients with multimorbidity used pain medication for LBP at 12 months follow-up compared to those without chronic disease (OR 2.36).

**Conclusions:**

Chiropractors should be aware that patients with LBP may suffer from multimorbidity with poor general health. Patients with multimorbidity also have poorer recovery from LBP than people without chronic disease and clinical follow-up may be indicated.

## Background

Multimorbidity is defined as the co-existence of two or more chronic conditions in an individual [1]. Estimates of the prevalence of multimorbidity in people presenting in primary care range from 13% in individuals aged 18 years and older to 95% in a population aged 65 years and older [2], and is projected to become more prevalent as the population ages [3]. Chronic musculoskeletal disorders include degenerative conditions, such as osteoarthritis, inflammatory rheumatic diseases, such as rheumatoid arthritis; fragility conditions, such as osteoporosis; and regional pain syndromes, such as low back pain (LBP), neck pain and fibromyalgia. A substantial proportion of people with musculoskeletal disorders live with multimorbidity [4–6]. People with multimorbidity often suffer from pain and functional limitations caused by musculoskeletal disorders in combination with their other chronic conditions [7]. Musculoskeletal disorders are present in patients with multimorbidity because of their high prevalence, shared risk factors, and shared pathogenic processes amongst other long-term conditions. Such musculoskeletal issues are often associated with decreased ability to work and lower quality of life [7].

Chiropractors are a part of primary care in Denmark and may therefore play an important role for patients with multimorbidity in regaining mobility and reducing pain. Chiropractors in Denmark are self-employed, and almost all clinics (94%) operate under the agreement between the Danish Chiropractor’s Association and the Danish Regions, and for most services, 20% is paid by the region and 80% by the patient directly or by a private health insurance [8]. In chiropractic practice, approximately two thirds of patients are treated for LBP [9, 10]. While it is known that LBP can be associated with co-morbidity [11, 12], it is unknown to what extend LBP is accompanied with chronic conditions and contribute to multimorbidity in chiropractic practice. Recognition, by health professionals, policymakers, non-profit organizations, and research funders, of the impact of multimorbidity in musculoskeletal health is essential when planning support for these patients. However, is it currently unknown to what extend chiropractors treat patients with multimorbidity and how this affects the patients and the clinical course of back pain.

The aim of this paper was therefore to determine the prevalence of multimorbidity among patients with LBP seeking care in chiropractic practice. Secondly, to investigate if the presence of multiple chronic conditions affects pain intensity, self-rated health, mental and physical health. Finally, to explore if individuals with multimorbidity have a different recovery for their LBP problem than individuals without multimorbidity.

## Methods

The Danish Chiropractic Low Back Pain Cohort (ChiCo) is a longitudinal observational cohort consisting of adults seeking care for LBP at 10 chiropractic clinics from the Central Denmark Region [8]. Only medium or large size clinics were invited to ensure adequate recruitment of study participants within a reasonable timeframe. Patients were recruited consecutively from November 2016 to December 2018 and followed until 12 months after enrolment. The Regional Research Ethics Committee determined that ChiCo did not require ethical approval because of the absence of a study-initiated clinical intervention. [Project-id: S-20162000-109]. All participants provided written informed consent prior to enrolment.

### Participants

Patients presenting in chiropractic clinics with a new episode of LBP with or without leg pain, aged 18 years or older, and who were able to complete electronic questionnaires in Danish were eligible for inclusion. A new episode of LBP was defined as consulting with LBP without this being a follow-up consultation as part of an already initiated course of treatment. Patients with a non-musculoskeletal cause of the LBP were excluded [8].

### Data collection

All patient-reported data (baseline and follow-up) were collected using electronic questionnaires in REDCap (https://www.project-redcap.org/). To minimize the time for completing questionnaires in the waiting room prior to the consultation, the baseline questionnaire for the ChiCo cohort was split into two separate questionnaires. The first baseline questionnaire (BL1) was completed by the patients before meeting the chiropractor, i.e., in the waiting area. The second baseline questionnaire (BL2) was sent to the patient by email to be completed on the same day after the consultation. Non-responders to BL2 received an electronic reminder after two days. Data on chronic diseases were reported in BL2. As such, only participants who completed both BL1 and BL2 were included in the current analysis. Follow-up questionnaires were collected at 2 weeks, 3 months, and 12 months after inclusion. The data collection procedures are described in detail elsewhere [8].

### Patient-reported information

Baseline information on included participants comprise demographics, education and work situation. (Table 1). Overall self-rated health was assessed using a single item with responses from 1, “excellent” to 5, “poor”. Participants then completed a question about their history of chronic conditions, namely: “Did a medical doctor ever tell you that you have or have had [list of 15 chronic conditions]?” The 15 chronic conditions were diabetes, osteoporosis, thrombus (in the hearth, brain or elsewhere), hypertension, psoriasis, rheumatoid arthritis, osteoarthritis, fibromyalgia, metabolic diseases, asthma, migraine, chronic inflammatory bowel disease, cancer, chronic obstructive pulmonary disease or bronchitis, and neurological disease. This method has previously been used in large Danish cohort studies such as in The Danish Twin Registry and applied in analyses relating to back pain in young [13] and older people [14] and radiating spinal pain as risk factor for work disability [15].

**Table 1:** Baseline characteristics

	Total	Missing	LBP^f^ Intensity < 7^a^	LBP Intensity ≥ 7^a^	*p*-value^e^
	(n = 2083)		(n = 1217)	(n = 825)	
Sex, n (%)		0			
Male	1165 (55.9)		719 (62.9)	424 (37.1)	0.0006
Female	918 (44.1)		498 (55.4)	401 (44.6)	
Age, mean (sd)	46.07 (13.50)	0	44.74 (13.61)	46.16 (13.28)	0.4852
BMI, n (%)		11			0.0122
≤ 25 kg/m^2^	853 (41.2)		521 (61.9)	321 (38.1)	
26–29 kg/m^2^	776 (37.5)		460 (60.9)	296 (39.2)	
≥ 30 kg/m^2^	443 (21.4)		232 (53.6)	201 (46.4)	
Smoker, n (%)		4			0.0036
Yes	336 (16.2)		171 (51.4)	162 (48.7)	
No, I have stopped smoking	711 (34.2)		427 (61.8)	264 (38.2)	
No, I have never smoked	1031 (49.6)		615 (60.7)	399 (39.4)	
Education n (%)		61			0.0878
None	313 (15.5)		179 (58.7)	126 (41.3)	
Vocational or short education	848 (42.0)		481 (57.7)	353 (42.3)	
Middle further education	553 (27.4)		320 (58.9)	223 (41.1)	
University degree	233 (11.5)		152 (66.7)	76 (33.3)	
Other	74 (3.7)		48 (67.6)	23 (32.4)	
Working, n (%)		32			0.1664
Yes	1683 (82.1)		973 (58.8)	681 (41.2)	
No	368 (17.9)		226 (62.8)	134 (37.2)	
Pain intensity^a^, mean (sd)					
Lower back pain (LBP)	6.70 (2.02)	41	5.45 (1.62)	8.55 (0.73)	–
Leg pain	3.04 (2.94)	44	2.51 (2.62)	3.84 (3.22)	< 0.0001
LBP duration, n (%)		13			< 0.0001
1–7 days	961 (46.4)		464 (22.9)	476 (23.5)	
1 week–3 months	743 (35.9)		479 (23.6)	255 (12.6)	
3–12 months	152 (7.3)		113 (5.6)	35 (1.7)	
12 + months	214 (10.3)		152 (7.5)	56 (2.8)	
Medication for LPB, n (%)		39			< 0.0001
Yes, prescription medication	355 (17.4)		142 (40.6)	208 (59.4)	
Yes, non-prescription medication	686 (33.6)		357 (53.6)	309 (46.4)	
No	1002 (49.1)		692 (70.2)	294 (29.8)	
Roland Morris Disability Questionnaire^b^, mean (sd)	12.72 (5.45)	155	10.92 (5.33)	15.40 (4.39)	< 0.0001
Mental health^c^, mean (sd)					
Depressed	3.02 (3.96)	31	2.70 (2.76)	3.52 (3.16)	< 0.0001
Stressed/anxious	3.85 (2.95)	25	3.62 (2.85)	4.22 (3.08)	< 0.0001
Muskuloskeletal pain, n (%)					
Head	726 (34.9)	0	415 (57.7)	304 (42.3)	0.2068
Neck	910 (43.7)	0	519 (58.1)	375 (42.0)	0.2152
Chest	116 (5.6)	0	65 (56.0)	51 (44.0)	0.4232
Stomach	240 (11.5)	0	130 (54.2)	110 (45.8)	0.0689
Shoulders	850 (40.8)	0	496 (59.5)	337 (40.5)	0.9788
Elbows	147 (7.1)	0	86 (59.7)	58 (40.3)	0.9709
Arms	217 (10.4)	0	117 (55.2)	95 (44.8)	0.1688
Hands	235 (11.3)	0	122 (52.8)	109 (47.2)	0.0261
Hips	552 (26.5)	0	306 (56.9)	232 (43.1)	0.1368
Knees	558 (26.8)	0	314 (57.4)	233 (42.6)	0.2258
Legs	502 (24.1)	0	287 (58.6)	203 (41.4)	0.6022
Feet	371 (17.8)	0	219 (60.5)	143 (39.5)	0.6946
Other	90 (4.3)	0	65 (73.9)	23 (26.1)	0.0052
No pain	337 (16.2)	0	205 (62.1)	125 (37.9)	0.3040
Self-rated general health, n (%)		639			0.0037
Excellent	221 (15.3)		135 (63.1)	79 (36.9)	
Very good	604 (41.9)		369 (62.1)	225 (37.9)	
Fine	378 (26.2)		217 (58.3)	155 (41.7)	
Fair	204 (14.1)		104 (51.5)	98 (48.5)	
Poor	36 (2.49)		13 (37.1)	22 (62.9)	
Physical ressources^d^, mean (sd)					
Aerobic fitness	5.17 (1.86)	58	5.27 (1.80)	4.99 (1.93)	0.0007
Muscular strength	5.86 (1.71)	57	5.88 (1.63)	5.81 (1.81)	0.3778
Endurance	5.71 (1.90)	59	5.78 (1.83)	5.61 (2.00)	0.0548
Flexibility	5.00 (1.95)	60	5.04 (1.87)	4.92 (2.05)	0.1544
Balance	5.65 (1.87)	60	5.66 (1.79)	5.64 (1.99)	0.7751

^a^Pain was rated on numeric rating scales (NRS) 0–10 (higher score indicating worse pain)

^b^The Roland Morris Disability Questionnaire 0–23 (higher score indicating more disability)

^c^Mental health was assessed by the Örebro Musculoskeletal Pain Screening Questionnaire 0–10 (higher score indicating worse mental health)

^d^Physical resources was assessed on a visual analog scale from 1 (poor) to 9 (excellent)

^e^*p*-value of a chi-squared test (categorical variables) or t-test (continuous variables)

Multimorbidity was defined as the presence of two or more of these chronic conditions [1].

Pain intensity was rated progressively 0–10 on numeric rating scales [NRS] for LBP and leg pain separately [16], and participants were asked about duration of current episode, use of pain medications for LBP, and number of days with LBP within the past year. Participants also reported recent musculoskeletal pain other than LBP. Physical function was measured by assessing activity limitation due to LBP [Roland Morris 23-item Disability Questionnaire [RMDQ]] [16, 17], and self-perceived physical resources including physical fitness, muscle strength, endurance, flexibility and balance [18]. Information collected about mental health included feeling depressed or stressed as assessed by the Örebro Musculoskeletal Pain Screening Questionnaire [19].

### Data analysis

Frequencies of demographic variables at baseline were grouped by intensity of LBP (NRS ≥ 7 vs. < 7) and compared using Chi-squared tests. To facilitate comparisons, we dichotomized each outcome variable. Cut points for dichotomization were chosen based on the distribution of the data or according to the scoring of each instrument. High intensity of LBP was defined as NRS ≥ 7 [20]. This intensity of LBP cut-off was used to report frequencies of demographic variables at baseline and compared using Chi-squared tests. Low self-rated health was defined as a score of ≥ 4 indicating fair or poor health. The thresholds for poor mental and physical health and ability to do everyday activities were set at the poorest quartile in the dataset. Logistic regression models were fitted and odds ratios (ORs) and corresponding 95% confidence intervals (95% CIs) were reported adjusting for age, BMI, sex, smoking status, employment status, cohabitation, education, pain duration, and presence of other musculoskeletal pain.

To evaluate differences in recovery from the LBP problem, we evaluated the RMDQ score and use of pain medication across the four time points grouped by chronic disease variables. For the RMDQ score, we reported differences in mean score, with 95% confidence intervals and *p*-values, from a linear regression model fitted with generalized estimating equation (GEE) to account for repeated observations on the same individuals and the weighting procedure described below. For pain medication, we reported odds ratios with 95% confidence intervals and *p*-values from a logistic regression model fitted with GEE. Weights were used to adjust for differential dropout [21]. The weights were calculated by inverse probability and estimated using age, BMI, number of children, sex, smoking status, employment status, cohabitation, chronic disease variables, education, pain duration, presence of other musculoskeletal pain, chiropractic clinic, treatment received by other practitioners (i.e., physical therapist), self-rated health, and physical resources. All these analyses were performed adjusting for age, BMI, sex, smoking status, employment status, cohabitation, education, pain duration, and presence of other musculoskeletal pain. All analyses were carried out in the SAS 9.4 statistical package. The significance level was set at *p* < 0.01.

## Results

**Fig. 1:**
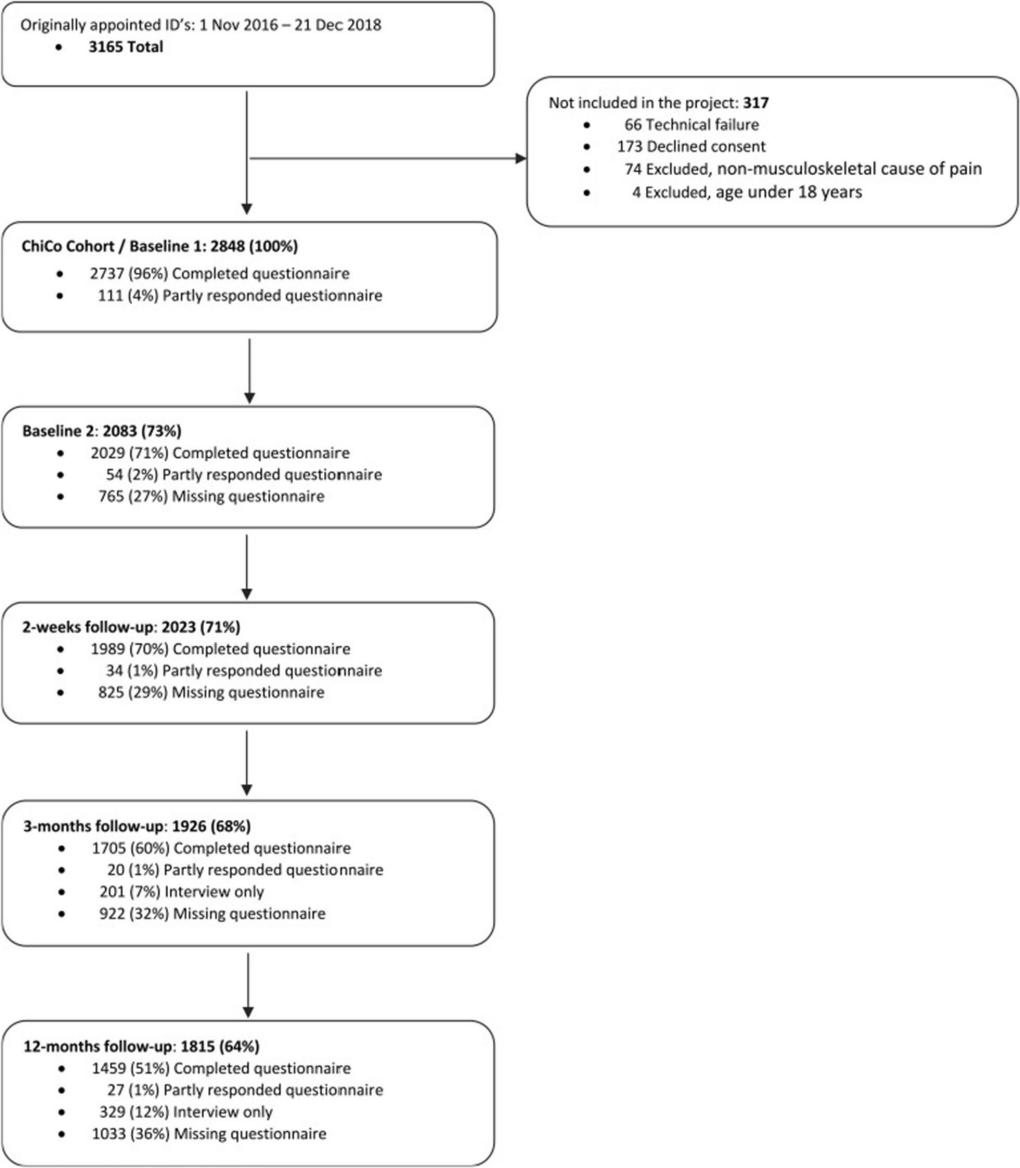
Flow chart

A total of 2848 patients consented of whom 2083 (74%) completed both the BL1 and BL2 and were included in the analysis. Of the consenting patients 71%, 68% and 64% responded to follow-up questionnaires at 2 weeks, 3 months and 12 months. (Fig. 1: Flow chart). Most participants were males (56%) with an average age of 46 (SD 13) years (Table 1). The most common chronic conditions were high blood pressure (19%) and osteoarthritis (15%) (Table 2).

**Table 2:** Prevalence of chronic disease among patients with LBP at baseline (n = 2083)

Disease	Prevalence (%)
Diabetes	73 (3.5)
Osteoporosis	33 (1.6)
Stroke/heart attack	81 (3.9)
High blood pressure	395 (19.0)
Psoriasis	105 (5.0)
Rheumatoid arthritis	34 (1.6)
Osteoarthritis	305 (14.6)
Fibromyalgia	9 (0.4)
Metabolic diseases	97 (4.7)
Asthma	180 (8.6)
Migraine	182 (8.7)
Chronic inflammatory bowel disease	27 (1.3)
Cancer	79 (3.8)
COPD or chronic bronchitis	42 (2.0)
Neurological disease	16 (0.8)
No disease	890 (42.7)

**Fig. 2:**
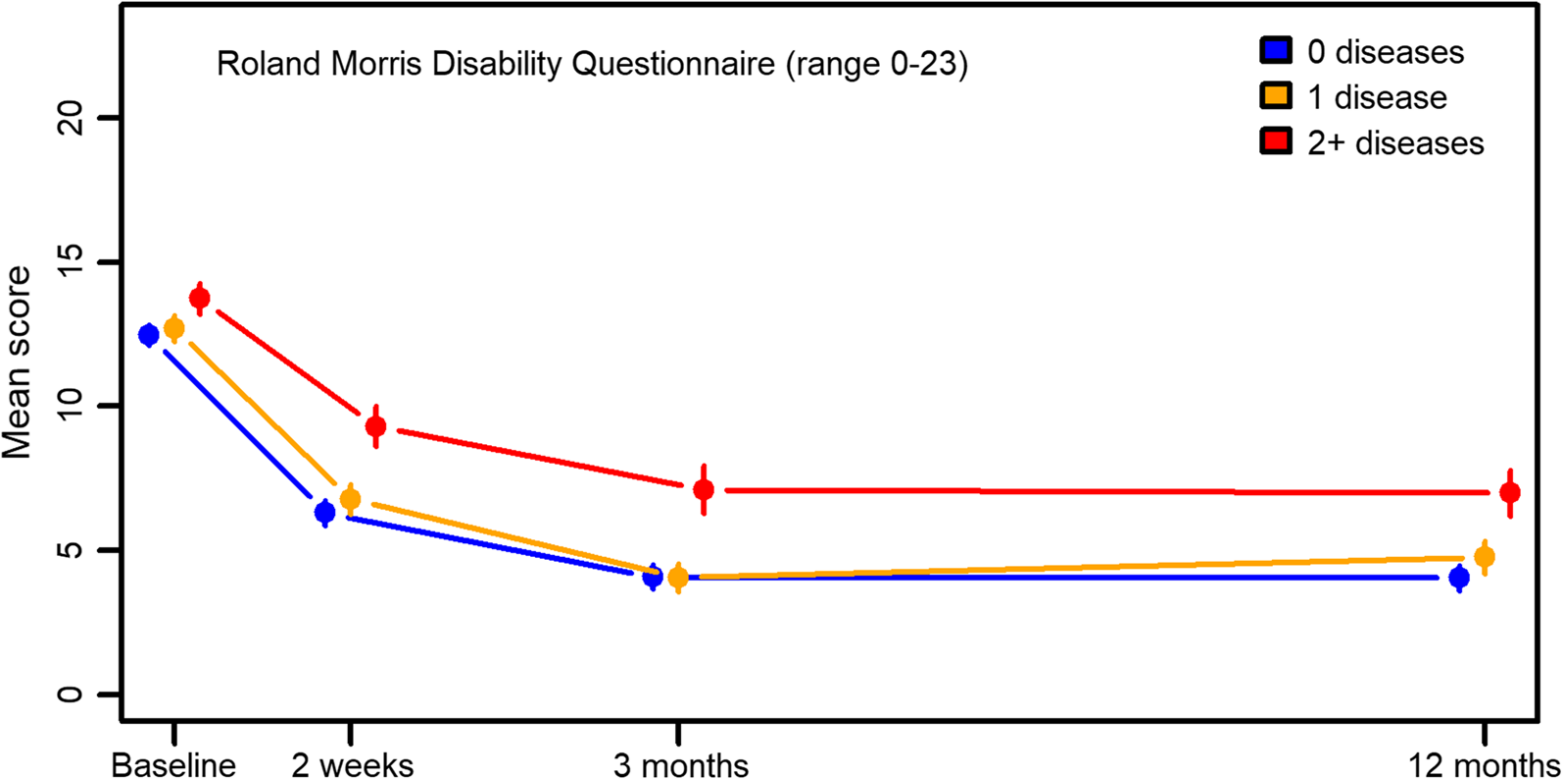
Roland Morris 23-item Disability Questionnaire (RMDQ) score

**Fig. 3:**
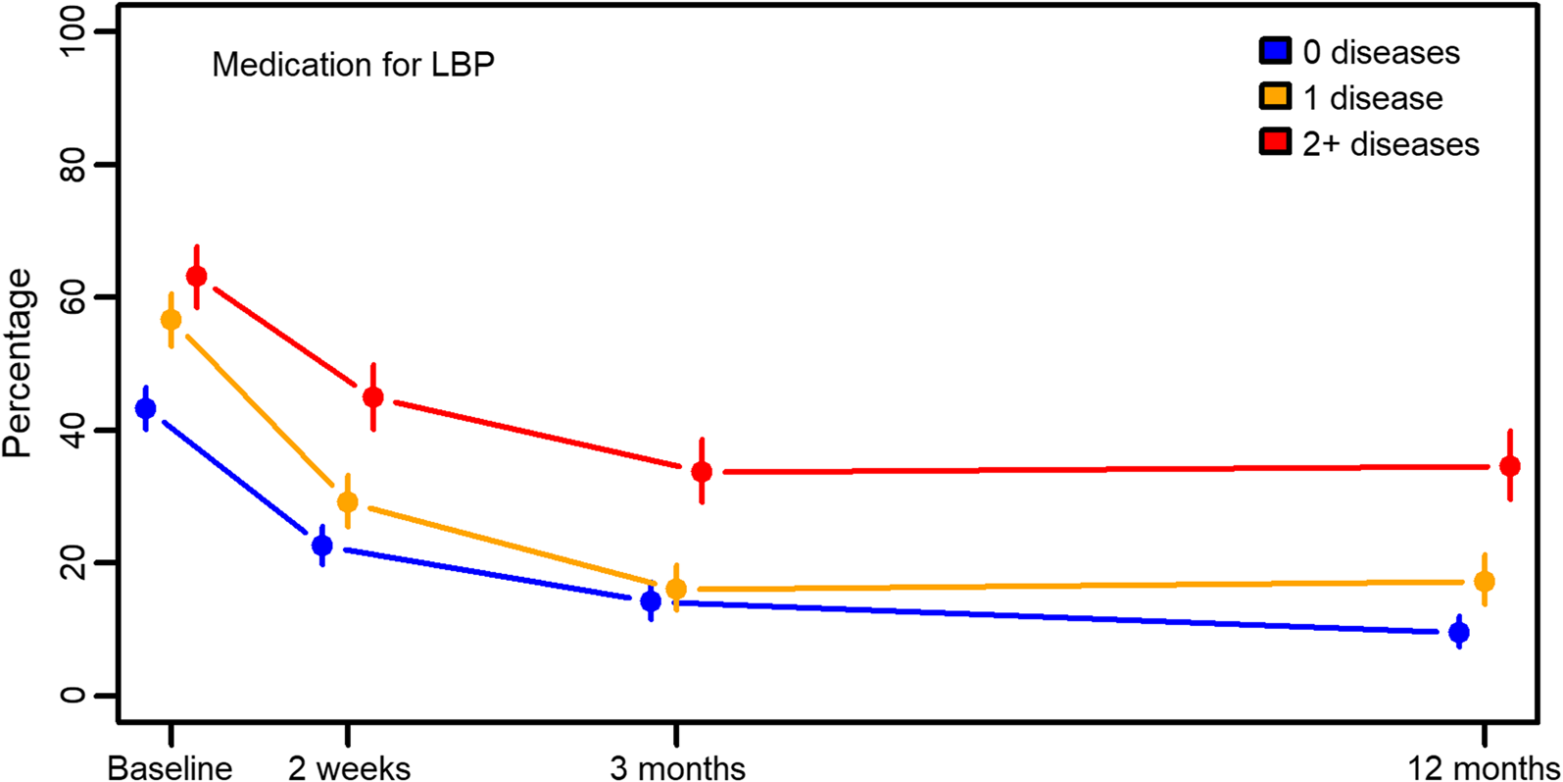
Medication for low back pain (LBP) k pain (LBP).

Of the total sample, 1024 (49%) participants reported to have at least one chronic condition and 421 (20%) had multimorbidity (≥ 2 chronic conditions) (Table 3). At baseline, 825 (40%) participants reported high LBP intensity (Table 3). The presence of multimorbidity was associated with increased odds of poor self-rated health (OR 2.13, 95% CI 1.46; 3.11), poor physical fitness (OR 1.79, 95% CI 1.35; 2.38), poor muscular strength (OR 1.52, 95% CI 1.18; 1.96), poor endurance (OR 1.51, 95% CI 1.17; 1.96), and poor balance (OR 1.33, 95% CI 1,03; 1.72) (Table 3). Multimorbidity was not seen to be associated with depression or anxiety (Table 3). All participants regardless of pain intensity at baseline and presence of multimorbidity improved over time and fewer used pain medication (Figs. 2, 3). Patients with high LBP intensity combined with multimorbidity showed a poorer recovery than patients with high LBP intensity but without chronic diseases (mean difference 3.53, 95% CI 1.85; 5.20 at 12 months follow-up) (Table 4, adjusted). A clinical significant change is a score ranging from 2 to 4 points [22]. For patients with low LBP intensity, no such association was present (Table 4, adjusted). More patients with multimorbidity used pain medication for LBP at 12 months follow-up compared to those without chronic disease (OR 2.36 (1.52; 3.66)) (Fig. 3 and Table 4, adjusted).

**Table 3:** Impact on physical and mental health of chronic disease and multimorbidity at baseline

	Total	LBP intensity ≥ 7	Low SRH	Depressed	Stressed anxious	Poor physical fitness	Poor muscular strength	Poor endurance	Poor flexibility	Poor balance
N (%)	2083 (100.00)	825 (40.40)	240 (16.63)	513 (25.00)	486 (23.62)	649 (32.07)	848 (41.88)	918 (45.38)	769 (38.03)	1029 (50.89)
Number of diseases	N (%)	OR (95% CI)								
0	1059 (50.8)	(Ref)	(Ref)	(Ref)	(Ref)	(Ref)	(Ref)	(ref)	(Ref)	(Ref)
1	603 (29.0)	1.04 (0.83; 1.31)	1.25 (0.85; 1.85)	1.35 (1.05; 1.75)	1.40 (1.08; 1.82)	1.30 (1.01; 1.68)	0.93 (0.74; 1.16)	1.12 (0.89; 1.40)	1.11 (0.88; 1.39)	1.08 (0.87; 1.34)
2	271 (13.0)	1.21 (0.88; 1.67)	1.91 (1.19; 3.09)	1.31 (0.91; 1.89)	0.99 (0.67; 1.45)	1.82 (1.29; 2.57)	1.37 (1.00; 1.86)	1.49 (1.09; 2.04)	1.30 (0.95; 1.78)	1.34 (0.98; 1.82)
3	109 (5.2)	1.42 (0.89; 2.27)	3.34 (1.76; 6.32)	1.17 (0.69; 1.99)	1.25 (0.73; 2.15)	2.39 (1.46; 3.93)	1.51 (0.95; 2.37)	1.76 (1.10; 2.82)	1.09 (0.69; 1.73)	1.39 (0.88; 2.19)
4	29 (1.4)	1.16 (0.51; 2.66)	6.00 (1.99; 18.07)	0.70 (0.24; 2.00)	0.96 (0.33; 2.77)	6.06 (2.42; 15.18)	3.40 (1.41; 8.19)	2.70 (1.11; 6.58)	1.90 (0.85; 4.23)	1.43 (0.63; 3.24)
5+	12 (0.6)	0.60 (0.13; 2.73)	1.81 (0.36; 9.17)	0.11 (0.01; 1.25)	0.16 (0.02; 1.50)	0.88 (0.22; 3.37)	0.95 (0.27; 3.37)	1.40 (0.33; 5.88)	0.77 (0.21; 2.82)	5.18 (0.70; 47.94)
Multimorbidity (≥ 2 diseases)										
No	1662 (79.8)	(Ref)	(Ref)	(Ref)	(Ref)	(Ref)	(Ref)	(Ref)	(Ref)	(Ref)
Yes	421 (20.2)	1.22 (0.93; 1.59)	2.13 (1.46–3.11)	1.02 (0.76; 1.39)	0.86 (0.63; 1.18)	1.79 (1.35; 2.38)	1.52 (1.18; 1.96)	1.51 (1.17; 1.96)	1.20 (0.93; 1.56)	1.33 (1.03; 1.72)
Missing	0	41	640	31	25	59	58	60	61	61

**Table 4:** Roland Morris Questionnaire score and pain medication in relation to multimorbidity and chronic disease (adjusted)

	Baseline		2 weeks		3 months		12 months	
	Difference (95%CI)	*p*-value	Difference (95%CI)	*p*-value	Difference (95%CI)	*p*-value	Difference (95%CI)	*p*-value
*Roland Morris Questionnaire*								
Total								
0 diseases	(Ref)		(Ref)		(Ref)		(Ref)	
1 disease	− 0.18 (− 0.74; 0.39)	0.535	0.16 (− 0.54; 0.86)	0.653	− 0.33 (− 1.11; 0.45)	0.411	0.49 (− 0.37; 1.35)	0.259
2+ diseases	− 0.11 (− 0.79; 0.58)	0.760	1.42 (0.59; 2.25)	0.001	1.30 (0.31; 2.29)	0.010	1.46 (0.39; 2.52)	0.007
LBP intensity < 7								
0 diseases	(Ref)		(Ref)		(Ref)		(Ref)	
1 disease	0.21 (− 0.53; 0.96)	0.575	− 0.34 (− 1.19; 0.52)	0.440	− 0.75 (− 1.70; 0.19)	0.118	− 0.08 (− 1.07; 0.90)	0.866
2+ diseases	0.74 (− 0.18; 1.67)	0.117	1.04 (− 0.05; 2.13)	0.063	0.12 (− 1.13; 1.37)	0.849	0.38 (− 0.96; 1.72)	0.581
LBP intensity ≥ 7								
0 diseases	(Ref)		(Ref)		(Ref)		(Ref)	
1 disease	− 1.03 (− 1.84; − 0.22)	0.013	1.20 (− 0.01; 2.41)	0.051	0.61 (− 0.67; 1.90)	0.3493	1.62 (0.18; 3.07)	0.0274
2+ diseases	− 1.57 (− 2.54; − 0.58)	0.002	2.38 (1.08; 3.69)	< .001	3.24 (1.71; 4.77)	< .001	3.53 (1.85; 5.20)	< .001

Pain medication for LBP								
	OR (95%CI)	*p*-value	OR (95%CI)	*p*-value	OR (95%CI)	*p*-value	OR (95%CI)	*p*-value
Total								
0 diseases	(Ref)		(Ref)		(Ref)			
1 disease	1.45 (1.16; 1.82)	0.001	0.79 (0.59; 1.05)	0.110	0.67 (0.46; 0.99)	0.043	1.19 (0.78; 1.81)	0.428
2+ diseases	1.35 (1.03; 1.79)	0.032	1.15 (0.83; 1.57)	0.4004	1.42 (0.95; 2.11)	0.0890	2.36 (1.52; 3.66)	< .001
LBP intensity < 7								
0 diseases	(Ref)		(Ref)		(Ref)		(Ref)	
1 disease	1.44 (1.07; 1.94)	0.018	0.71 (0.48; 1.06)	0.0940	0.64 (0.38; 1.06)	0.0853	1.21 (0.66; 2.22)	0.529
2+ diseases	1.44 (0.99; 2.09)	0.054	1.07 (0.70; 1.65)	0.7541	1.32 (0.78; 2.24)	0.2966	2.43 (1.31; 4.51)	0.005
LBP intensity ≥ 7								
0 diseases	(Ref)		(Ref)		(Ref)		(Ref)	
1 disease	1.38 (0.95; 2.03)	0.095	0.91 (0.58; 1.44)	0.692	0.77 (0.41; 1.41)	0.394	1.13 (0.60; 2.14)	0.710
2+ diseases	1.13 (0.72; 1.78)	0.594	1.44 (0.87; 2.37)	0.1543	2.13 (1.12; 4.02)	0.0203	2.80 (1.44; 5.44)	0.002

## Discussion

This is the first manuscript to report on the prevalence and impact of multimorbidity among patients with LBP treated in chiropractic practice. Approximately 20% of patients had multimorbidity, which was associated with poorer self-rated health, physical fitness, muscular strength, endurance, and balance at baseline. In addition, patients with multimorbidity and high LBP intensity had poorer recovery in terms of disability in everyday activities and continued need for pain medication.

In a systematic review from primary care, prevalence rates of multimorbidity of 20–50% were found for patients of similar age as those included in our study. Thus for most settings in primary care, the prevalence of multimorbidity was higher than that observed in the current study (20%) [2]. For people seeking care from chiropractors specifically, not much is known about patterns of multimorbidity. In Australia, Charity et al. studied profiles of patients seeking care from chiropractors for multiple reasons and found that 24% reported circulatory, 24% reported endocrine and metabolic, and 12% reported respiratory comorbidities [23]. These numbers are somewhat higher than ours, which might be explained by the fact that we included only patients presenting with a new episode of LBP and not consecutive patients such as in the Australian study. In Denmark, profiles of people seeking care for LBP in general practice and chiropractors have been compared, and chiropractic patients were fund to be younger, more often males and better on all LBP disease-related parameters [24]. De Luca et al. also found that older Australians who sought care from chiropractors had significantly fewer comorbidities compared to those who sought care from general practitioners [25]. The observed general good health of patients in chiropractic practice may explain the relatively low prevalence of multimorbidity in our study.

Generally, people with multimorbidity and co-occurring musculoskeletal pain report higher levels of disability [12, 26] as well as more mental health problems [27], physical inactivity and obesity [28]. Therefore, it is not surprising that those with multimorbidity report poorer self-rated health in our study. The observed poorer outcomes among patients in this cohort with multimorbidity and high pain levels is to be expected. It is well-known that people with back pain and other musculoskeletal conditions and multimorbidity respond less well to treatments including pharmacological and non-pharmacological interventions as well as surgical treatment [29]. Therefore, the challenge for chiropractors—and indeed for all healthcare professionals who treat people with LBP—is to not view LBP as an isolated regional pain condition but one manifestation of poor health that for many include pain at other body sites as well as disease in other body systems [6, 29]. Chiropractors in Denmark generally have a broad approach to care suited to patients with more complex problems such as people with multimorbidity. It includes patient education, exercise facilities, and promotion of physical activity in addition to more traditional manual treatment. Of the chiropractors in Denmark, 45% work in a multidisciplinary setting with physical therapists [30, 31]. In addition, more than 90% communicate electronically with the broader healthcare system including with general practitioners [31]. In their contract, the chiropractors have special obligations to inform the general practitioner about patients with complex problems, but the quality of this knowledge exchange is unknown.

### Strengths and limitations

The cohort is the largest and most comprehensive cohort of patients with LBP seeking care from chiropractors. The participation rates at both short- and long-term follow-up were satisfactory, and our drop-out analyses indicate minimal attrition bias over the one year [8]. The procedures in the study were pre-tested in a feasibility study and validated scales for the outcomes were chosen.

Only medium or large size clinics were invited. It is, however, possible that only recruiting from larger clinics may have introduced some bias into the sample. Finally, it is a limitation that the chronic conditions were self-reported without validation in for example medical records. Some rare chronic conditions may not be covered by the list.

## Conclusions

Approximately 20% of patients with LBP in this cohort of Danish chiropractric practices have multimorbidity. Patients with multimorbidity reported higher pain levels, poorer self-rated health, and poorer physical functioning compared to those without multimorbidity. The LBP problem improved with time both for those with multimorbidity and those without. However, patients with high baseline pain levels and multimorbidity, had poorer recovery in terms of self-reported back-related disability. Furthermore, patients with multimorbidity had a continued use of pain medications for LBP compared to those without chronic disease. Chiropractors should be aware that patients with high levels of LBP and multimorbidity have a poorer recovery than patients without chronic disease, and clinical follow-ups may be indicated. The challenge of multimorbidity to chiropractors is not to view LBP as an isolated pain condition but rather it may be a part of a complex of diseases.

## Data Availability

The full data set are available. Contact: Professor Jan Hartvigsen, Department of Sports Science and Clinical Biomechanics, University of Southern Denmark, Odense, Denmark e-mail: jhartvigsen@health.sdu.dk.

## Abbreviations

LBP: Low back pain
ChiCo: The Danish Chiropractic low back pain Cohort
RMDQ: The Roland Morris Disability Questionnaire
BL1: The first baseline questionnaire
BL2: The second baseline questionnaire
NRS: Numeric rating scale
GEE: Generalized estimating equation
BMI: Body mass index
